# Role of gut microbiota in bempedoic acid against hyperlipidemia: a new candidate target for bempedoic acid on the therapeutic regulation

**DOI:** 10.3389/fphar.2025.1584273

**Published:** 2025-06-03

**Authors:** Wei-Jian Zhang, Jian Wang, Zi-Qi Ouyang, Sen-Rong Luo, Zhi-Kai Chen, Yuan-Kang He, Mu-Jin Luo, Xu-Xing Liao

**Affiliations:** ^1^ Department of Neurosurgery, First People’s Hospital of Foshan, Foshan, Guangdong, China; ^2^ Department of Translational Medicine Research Institute, First People’s Hospital of Foshan, Foshan, Guangdong, China; ^3^ Department of Neurosurgery and Advanced National Stroke Center, Foshan Sanshui District People’s Hospital, Foshan, Guangdong, China; ^4^ First Clinical Medical College, Guangdong Medical University, Zhanjiang, Guangdong, China

**Keywords:** bempedoic acid (BA), hyperlipidemia, atherosclerosis, gut microbiota, serum metabolite

## Abstract

**Background:**

Bempedoic acid (BA), an oral synthetic dicarboxylic acid derivative, has been widely used to treat patients with hyperlipidemia who are intolerant to statins. Both clinical and preclinical studies have reported the protective effects of BA against hyperlipidemia. However, the mechanisms of BA for hyperlipidemia treatment remain largely obscured. This study aimed to evaluate the effects of BA on hyperlipidemia and investigate the mechanism underlying its correlation with gut microbiota and serum metabolite regulation.

**Methods:**

Four-week-old male C57BL/6J mice were fed a high-fat diet (HFD) and treated with BA daily for 20 weeks. Biochemical changes of serum were assessed, covering lipid metabolism, inflammation, and endothelial function. Moreover, 16S rRNA sequencing of gut microbiota as well as untargeted metabolomics analysis of serum were performed.

**Results:**

The results showed that BA exerted potent efficacy against hyperlipidemia by attenuating serum lipid profiles, inhibiting vascular inflammation, and improving endothelial function. 16S rRNA sequencing revealed that BA improved the disorder of gut microbiota biodiversity. Specifically, BA significantly enriched the abundance of *Akkermansia*, *Bacteroides*, *Roseburia*, *Faecalibacterium* and *Uncultured_bacterium_f_Muribaculaceae*, which were closely associated with BA’s therapeutic effects. Serum metabolomics analysis indicated that BA recovered the disturbances of serum metabolic phenotypes. A total of 20 differential metabolites were significantly regulated by BA treatment, suggesting that BA might exert effects by ameliorating relevant metabolic pathways, including bile acid biosynthesis, glycerophospholipid metabolism, β-oxidation of fatty acids and sphingolipid metabolism.

**Conclusion:**

Taken together, the present study demonstrated that BA could inhibit the development of hyperlipidemia, and its protective effects may be related to alterations in gut microbiota composition and changes in serum metabolite abundances.

## Graphical Abstract

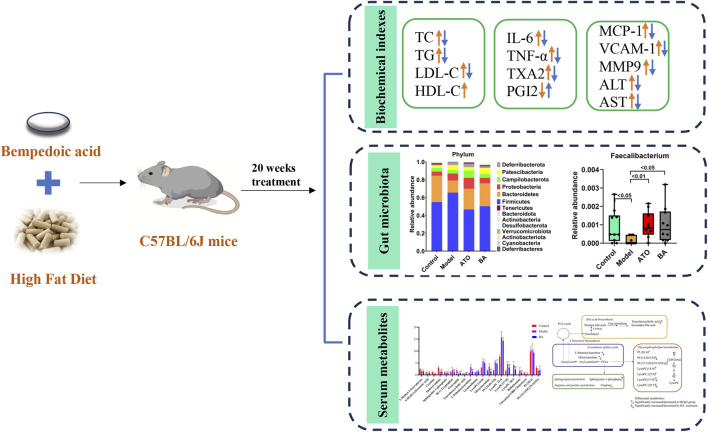

## 1 Introduction

Hyperlipidemia is a common metabolic disorder among middle-aged and elderly populations, characterized by abnormally elevated blood lipid levels. It is a significant modifiable risk factor for atherosclerotic cardiovascular disease (Skoumas et al., 2025; Zhang and Yao, 2025; Stewart et al., 2020). In recent years, a growing body of evidence has revealed that the composition of gut microbiota, together with its metabolites, are closely linked to the development and aggravation of hyperlipidemia (Jia et al., 2021; Lv et al., 2023). Notably, an increased *Firmicutes*/*Bacteroidetes* (F/B) ratio is widely regarded as a key indicator of gut microbiota dysbiosis in hyperlipidemia (Lu et al., 2022). Specific bacterial genera such as *Akkermansia* and B*acteroides* have been reported to be associated with the development of hyperlipidemia (Hasani et al., 2021; Yoshida et al., 2018). In addition, gut microbiota-related metabolites such as lysophosphatidylcholines (LysoPCs), long-chain acylcarnitine, and bile acids have also been discovered to be involved in the regulation of hyperlipidemia (He and You, 2020).

Bempedoic acid (BA) is a novel small-molecule oral hypolipidemic drug, which was developed by the American biopharmaceutical company Esperion (Nissen S. A.-O. et al., 2023). Both BA monotherapy and its combination with ezetimibe received marketing approval from the U.S. Food and Drug Administration (FDA) and the European Medicines Agency (EMA) in February 2020 (Marrs and Anderson, 2020; Agha et al., 2021). BA functions by inhibiting cholesterol synthesis in the liver to reduce LDL-C level in the blood (Ruscica et al., 2022). Several studies have demonstrated that BA can protect against hyperlipidemia and atherosclerosis (Biolo et al., 2022; Tummala et al., 2022). Moreover, clinical trials have confirmed that BA exhibits a lipid-lowering efficacy comparable to statins in certain patient populations (Kim et al., 2022; Nissen S. E. et al., 2023).

Recent research has increasingly focused on how lipid-lowering drugs, such as statins, modulate gut microbiota and associated metabolites in hyperlipidemia (Sun et al., 2022). However, to date, there has been a significant gap in understanding the impact of BA on the gut microbiota. Elucidating how BA might beneficially modify the gut microbiota and its metabolic output has become increasingly important, as this could reveal novel mechanisms of action or identify biomarkers for treatment response. Therefore, this study aimed to further investigate the therapeutic effects and mechanisms of BA in hyperlipidemia, with a specific focus on its regulatory role in gut microbiota composition and associated metabolite profiles, to clarify its comprehensive role in hyperlipidemia management.

## 2 Materials and methods

### 2.1 Drugs and reagents

BA (Chemical Abstracts Service (CAS) NO: 738606-46-7) was purchased from MedChemExpress (Monmouth Junction, NJ, United States). Atorvastatin (ATO) was purchased from Huirui Pharmacy Co., Ltd. Rodent standardized diet and high-fat diet (HFD) containing 21% fat and 1.5% cholesterol were purchased from Guangdong Medical Experimental Animal Center (Foshan, China). MS grade methanol and acetonitrile were purchased from Thermo Fisher Scientific Inc. (Fair Lawn, United States). Deionized water was purified by the Milli-Q system (Millipore, Merck KGaA, Darmstadt, Germany) and filtered through 0.22 μm membrane filter prior to use.

### 2.2 Animal model

All the experimental procedures were carried out in accordance with the National Institutes of Health guide for the care and use of Laboratory animals and were approved by the Ethics Committee of Guangdong Medical Laboratory Animal Center. During the experiment, appropriate procedures were taken to minimize the harm to the animals.

Four-week-old male C57BL/6J mice were obtained from Guangdong Medical Experimental Animal Center (Certification No. SCXK-(Yue) 2018-0002, Quality Qualification Certificate No. 44007200080586) and were raised in the houses of Guangdong Medical Laboratory Animal Center. The mice were housed in a specific pathogen-free environment with a room temperature at 23°C∼26°C, relative humidity of 50%∼70%, 12 h light/dark cycle, and freely acquirable food and water. Experiments began after the mice adapted to the new environment for 1 week. The C57BL/6J mice were randomly divided into four groups (n = 10 in each group) and received the following treatment for 20 weeks: (1) BA group: C57BL/6J mice were fed with HFD and given 10 mg/kg body weight BA by gavage once daily. (2) ATO group: C57BL/6J mice were fed with HFD and given 10 mg/kg body weight ATO by gavage once daily. (3) Model group: C57BL/6J mice were fed with HFD and given with the same volume of saline by gavage once daily. (4) Control group: C57BL/6J mice were fed a standardized normal diet and supplied with the same volume of saline. At the end of the experiment, all mice were sacrificed under anesthesia with intraperitoneal injection of pentobarbital sodium (150 mg/kg body weight) after overnight fasting followed by collection of blood samples. The serum was obtained from blood by centrifugation for 15 min at 1,000 × g and was stored at −80°C until the following analysis. Fecal samples were collected from each animal, transferred into sterile conical tubes, and immediately frozen in liquid nitrogen and then stored at −80°C until further analysis.

### 2.3 Measurement of blood biochemical indices

Serum levels of total cholesterol (TC), total glyceride (TG), low-density lipoprotein cholesterol (LDL-C), high-density lipoprotein cholesterol (HDL-C), interleukin-6 (IL-6), tumor necrosis factor-α (TNF-α), aspartate transaminase (AST), alanine transaminase (ALT), thromboxane A2 (TXA2), prostacyclin (PGI-2), monocyte chemoattractant protein-1 (MCP-1), vascular cell adhesion molecule-1 (VCAM-1) and matrix metalloproteinase-9 (MMP-9) were determined using kits (Nanjing Jiancheng Bio-engineering Institute, Nanjing, China) according to manufacturer’s instructions.

### 2.4 Extraction and detection of fecal genomic DNA and 16S rRNA gene sequencing

The PowerSoil DNA Isolation kit (MO BIO Laboratories, United States) was used to extract the fecal DNA. The V1-V9 variable regions of the 16S rRNA genes from bacterial and archaeal samples were amplified via PCR using the primer 27F (5′-AGRGTTTGATYNTGGCTCAG-3′) and 1492R (5′-TASGGHTACCTTGTTASGACTT-3′), which included adapter sequences and barcode sequences. After PCR amplification, sequencing was conducted on the Illumina HiSeq 2,500 platform (Illumina, Inc., San Diego, CA, United States) by Biomarker Technologies Co. Ltd. (Beijing, China). The sequencing data were deposited in the National Center for Biotechnology Information (NCBI) Sequence Read Archive (accession number SRP563800).

### 2.5 Sequencing data analysis

The Quantitative Insights Into Microbial Ecology (QIIME) version 1.8.0 was utilized to conduct the analysis of the 16S rRNA gene sequence data (Caporaso et al., 2010). The operational taxonomic units (OTUs) were selected by clustering sequences with a similarity of >97% using Usearch algorithm (Edgar, 2013). The Greengenes 16S rRNA gene database with a confidence threshold of 70% was used to obtain the relative abundance of each OTU and the taxonomy of each 16S rRNA gene sequence. 16S rRNA Sequencing depth data in all samples was shown in Supplementary Table S1. Alpha diversity indexes were calculated by mothur v1.31.2 software, which including the Chao 1 index, ACE index and Shannon index. The Nonmetric multidimensional scaling (NMDS) based on Bray Curtis distance algorithm was used to evaluate the beta diversity. The significant changed bacteria were tested by Metastats (http://metastats.cbcb.umd.edu/). Correlations among abundances of gut microbiota, blood biochemical indices, and serum metabolites were identified using Spearman’s correlation and visualized by heatmap. The *p*-values were corrected by *p*.adjust in the R (v3.0.3) with the method of Benjamini-Chochberg.

### 2.6 Untargeted metabolomics analysis of serum samples

#### 2.6.1 Sample preparation

The mice serum was taken out from the refrigerator at −80°C, defrosted at 4°C, and vortexed for 10 s 400 μL cold methanol/acetonitrile (1:1,v/v) containing 20 μg/mL myristo-D27 (internal standard) was added to 100 μL serum. The mixture was then vortexed for 1 min, incubated at −20°C for 1 h, centrifuged at 15,000 *g* for 20 min at 4°C. In the end, 200 μL supernatant was collected and 10 μL of the supernatant was injected for UFLC-Q-TOF-MS/MS analysis. Quality control (QC) samples, prepared by pooling 5 μL from each sample, were injected once every 8 samples during testing to monitor and adjusting system stability.

#### 2.6.2 UFLC-Q-TOF-MS/MS analysis

Untargeted metabolomics analysis of sample was carried out by UFLC-Q-TOF-MS/MS, ultra-fast liquid chromatography (Shimadzu Corp., Kyoto, Japan) coupled with quadrupole/time-of-flight mass spectrometry (Triple TOF 5600 plus, AB SCIEX, Foster City, CA, United States). The ACQUITY UPLC®HSS T3 column (1.8 μM, 2.1 × 100 mm) was applied to perform chromatographic separation at an oven temperature of 50°C. The mobile phase was 0.1% formic acid in deionized water (v/v) (A) and 0.1% formic acetonitrile (v/v) (B). The gradient elution procedure was as follows: 1% B (0-1.5 min), 1%-99% B (1.5–13 min), 99% B (13-16.5 min), 99%-1% B (16.5–17 min), 1% B (17–20 min) with the flow rate kept at 0.3 mL/min.

Mass spectrometry was conducted using an electrospray ionization (ESI) source. The ionspray voltage floating (ISVF) was set to 5,500 V in positive mode and 4,500 V in negative mode. The ion source temperature was maintained at 550°C.The mass range was from m/z 50–1,500 in both positive and negative mode. In both modes, the declustering potential (DP) was 80 V, collision energy was 30 V, and collision energy spread was 15 eV. The ion source gas 1 (GS1) and gas2 (GS2) were 55 psi, curtain gas (CUR) was 35 psi. Nitrogen was used as nebulizer and auxiliary gas. Data were acquired in information-dependent acquisition (IDA) mode using Analyst^®^ software (AB Sciex, Foster City, CA, United States).

### 2.7 Metabolomics data processing and analysis

The acquired raw data were converted into mzXML format using ProteoWizard. And then XCMS package in R programming software was performed for peak identification, extraction, comparison, filtering and missing peak supplement. The exported data were edited in Excel software, and result was organized into a two-dimensional data matrix, including retention time (RT), mass-to-charge ratio (m/z), samples, peak intensity and other information. The data were normalized using the intensity of the internal standard before chemometrics analysis. Then, the resulting data matrix was imported into SIMCA-P software version 14.1 (Umetrics, Umea, Sweden) to perform orthogonal partial least squares discriminant analysis (OPLS-DA). The metabolites with Variable importance value (VIP) > 1 and a value of *p* < 0.05 (significance test) among groups were considered as differential metabolites. The differential metabolites were further identified or tentatively characterized by mass fragmentation patterns in comparison with databases including Chemspider (www.chemspider.com) and HMDB (www.hmdb.ca). Pathway enrichment analysis was performed using MetaboAnalyst (http://www.metaboanalyst.ca/) and KEGG (http://www.genome.jp/kegg/) databases.

### 2.8 Statistical analysis

GraphPad Prism 8.0.2 (GraphPad Prism Software, La Jolla, CA, United States) and SPSS statistical software (version 22.0, SPSS. Inc., Chicago, United States) were used to perform statistical analysis. Data are presented as mean ± standard deviation (SD). Statistical significance for multiple group comparisons was assessed using one-way ANOVA followed by Dunnett’s multiple comparison tests. *p*-values less than 0.05 were considered to be statistically significant.

## 3 Results

### 3.1 BA inhibited the development of hyperlipidemia

**FIGURE 1:**
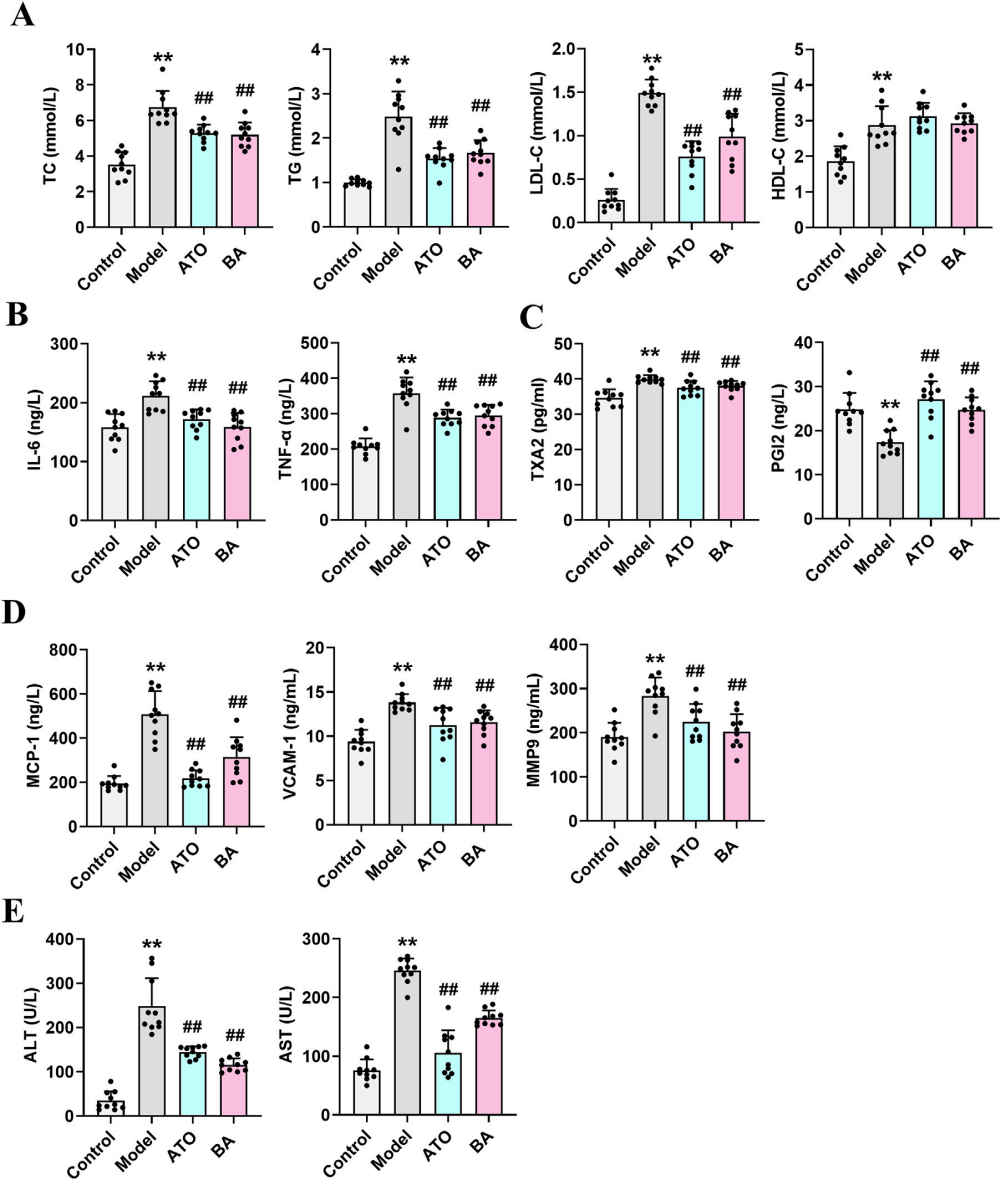
Effects of BA on serum biochemical parameters. **(A)** Serum levels of TC, TG, LDL-C and HDL-C; **(B)** Serum levels of IL-6 and TNF-α; **(C)** Serum levels of TXA2 and PGI2; **(D)** Serum levels of MCP-1, VCAM-1 and MMP9; **(E)** Serum levels of ALT and AST. Data are expressed as mean ± SD (n = 10 per group). Differences were assessed by one-way ANOVA with Dunnett’s method. ATO, Atorvastatin-treated group; BA, Bempedoic acid-treated group. Compared with Control group, ^*^ *p* < 0.05, ^**^ *p* < 0.01; Compared with Model group, ^#^ *p* < 0.05, ^##^ *p* < 0.01.

At the end of the study, lipid metabolism parameters were measured. As shown in Figure 1A, compared with the Control group, the model mice exhibited abnormal lipid metabolism with significantly elevated levels of serum TC, TG, and LDL-C, indicating that long-term HFD successfully induced hyperlipidemia. After drug administration, both ATO and BA effectively attenuated the upregulation of TG, TC, and LDL-C while increasing HDL-C levels (*p* < 0.05), demonstrating BA’s positive regulatory effects on blood lipid metabolism. Hyperlipidemia is often associated with chronic inflammation. Inflammatory cytokines play a critical role in the progression of hyperlipidemia to atherosclerosis. Compared with the Control group, the serum IL-6 and TNF-α levels were significantly elevated in the Model group (*p* < 0.01), indicating the long-term HFD induced systemic inflammation. After drug administration, ATO and BA significantly reduced IL-6 and TNF-α levels (*p* < 0.01) (Figure 1B), suggesting that BA could alleviate systemic inflammation during hyperlipidemia development. Hyperlipidemia is commonly accompanied by the endothelial dysfunction, which eventually leads to atherosclerosis. TXA2 and PGI2 are two arachidonic acid metabolites often used to characterize vascular endothelial function. A reduction in PGI2 and an elevation in TXA2 could promote the thrombogenesis (Viinikka, 1990; Niccoli et al., 2008). In this study, the level of PGI2 significantly decreased (*p* < 0.01) and the level of TXA2 significantly increased (*p* < 0.01) in the model mice, which suggested the endothelial dysfunction appeared. ATO and BA significantly increased the level of PGI2 and reduced the level of TXA2 (*p* < 0.01) (Figure 1C), indicating that BA effectively improved the endothelial function. Hyperlipidemia also triggers the release of chemokines, cytokines, and vascular growth factors. Compared with the Control group, the serum MCP-1, VCAM-1 and MMP9 levels were significantly increased in the Model group (*p* < 0.01). ATO and BA significantly reduced the levels of MCP-1, VCAM-1 and MMP9 (*p* < 0.05 or *p* < 0.01) (Figure 1D). Liver acts a central role in regulating both the synthesis and the catabolism of the plasma lipoproteins (Chen et al., 2024). In clinic, elevated blood lipid levels are commonly associated with the liver dysfunction. In the current study, compared with the Control group, the levels of ALT and AST in serum were significantly increased in the model group (*p* < 0.01), suggesting that the liver dysfunction appeared in HFD-fed mice. After administration, ATO and BA significantly lowered the serum ALT and AST levels (*p* < 0.01) (Figure 1E), indicating that BA could perform a protective effect on liver.

### 3.2 Regulation of BA on intestinal microecology in hyperlipidemia

**FIGURE 2:**
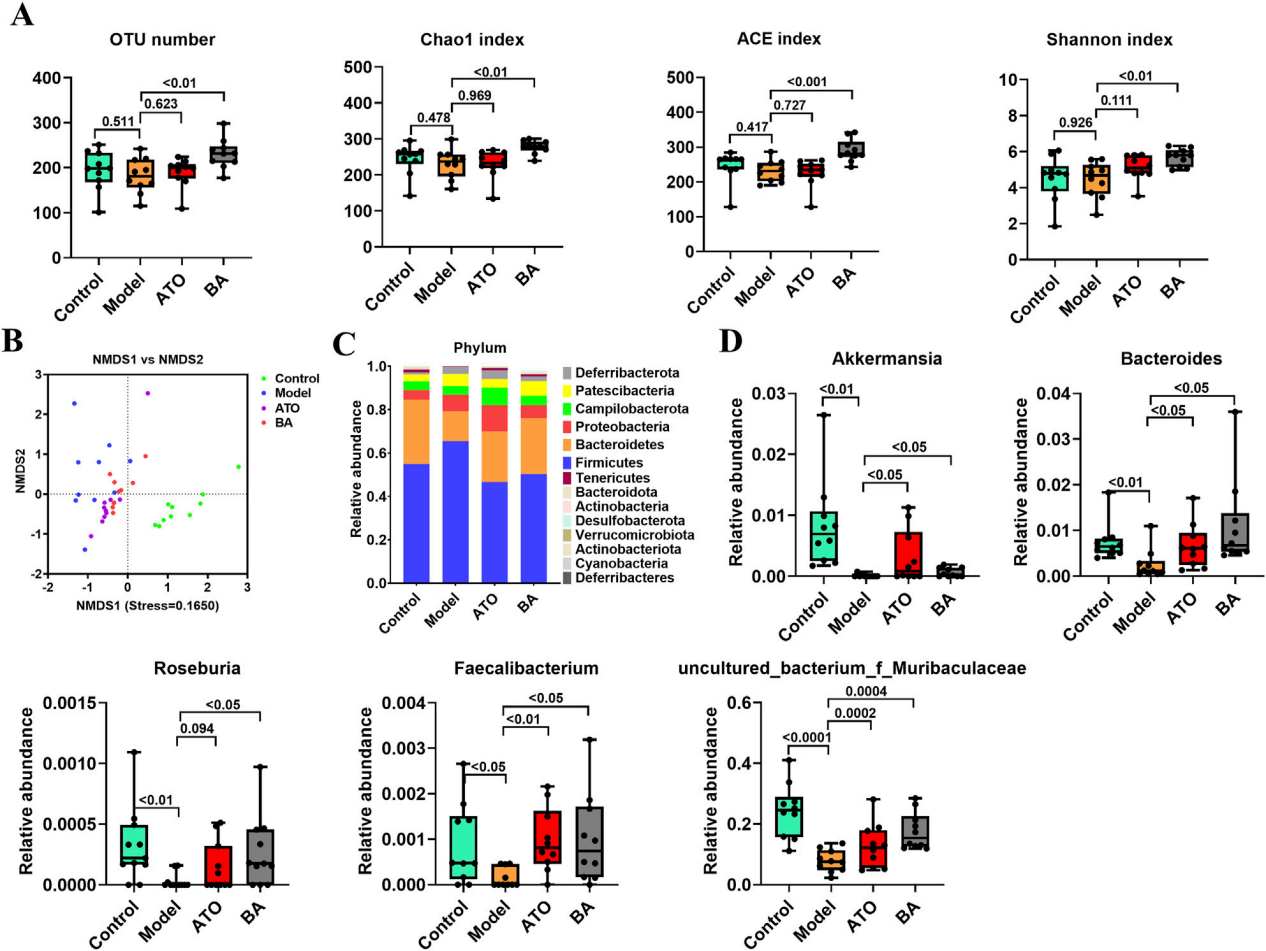
Effects of BA on gut microbiota disturbance. **(A)** Diversity of the gut microbiome assessed by OTU number, Chao1 index, ACE index and Shannon index among the test groups. **(B)** NMDS based on the Bray Curtis distance. Samples of the same color belong to the same group. The closer the samples are, indicating that the more similar the composition of the samples. **(C)** Relative abundances of gut microbiota at the phylum level. **(D)** Relative abundances of *Akkermansia*, *Bacteroides*, *Roseburia*, *Faecalibacterium* and *Uncultured_bacterium_f_Muribaculaceae*. Data are expressed as mean ± SD (n = 10 per group). Differences were assessed by one-way ANOVA with Dunnett’s method. ATO, Atorvastatin-treated group; BA, Bempedoic acid-treated group. A value of *p* < 0.05 was considered statistically significant.

To investigate the effect of BA on gut microbiota, Illumina HiSeq 2,500 platform was used to perform the 16S rRNA sequencing of fecal samples collected at the experimental endpoint. The observed OTUs, Chao 1 index, ACE index and Shannon index were calculated to estimate the alpha diversity. As shown in Figure 2A, compared with the Control group, the above indexes showed a significant reduction in the Model group. And, these four indexes were all significantly upregulated by BA treatment, which indicated that the long-term administration of BA could effectively improve the disorder of species diversity of gut microbiota. For beta diversity analysis, NMDS based on the Bray Curtis distance was used to visualize the dissimilarity in the compositions of the bacterial communities between different groups. As shown in Figure 2B, the distribution of samples in the Control group and the Model group was clearly separated, indicating that the community compositional structure significantly differed in the two groups. The distribution of samples in the BA group has a certain tendency to separate from the Model group, indicating that the BA treatment had a certain regulatory effect on the community structure of the HFD-fed mice.

In addition, the differences of the relative abundances of gut microbiota at different taxonomic levels were further analyzed. At the phylum level, the decreased abundance of *Bacteroidetes* and the increased abundance of *Firmicutes* were observed in the model group (Figure 2C), suggesting that the long-term HFD feeding could cause the increase of the ratio of the *Firmicutes* to *Bacteroidetes* (F/B ratio) in relative abundance. BA treatment reversed the increase of F/B ratio by increasing the abundance of *Bacteroidetes* and decreasing the abundance of *Firmicutes*. At the genus level, *Akkermansia*, *Bacteroides*, *Roseburia*, *Faecalibacterium* and *Uncultured_bacterium_f_Muribaculaceae* abundances were significantly reduced in model group compared with the control group. BA significantly increased the relative abundances of *Akkermansia*, *Bacteroides*, *Roseburia*, *Faecalibacterium* and *Uncultured_bacterium_f_Muribaculaceae* (Figure 2D).

### 3.3 Regulation of BA on serum metabolites in hyperlipidemia

**FIGURE 3:**
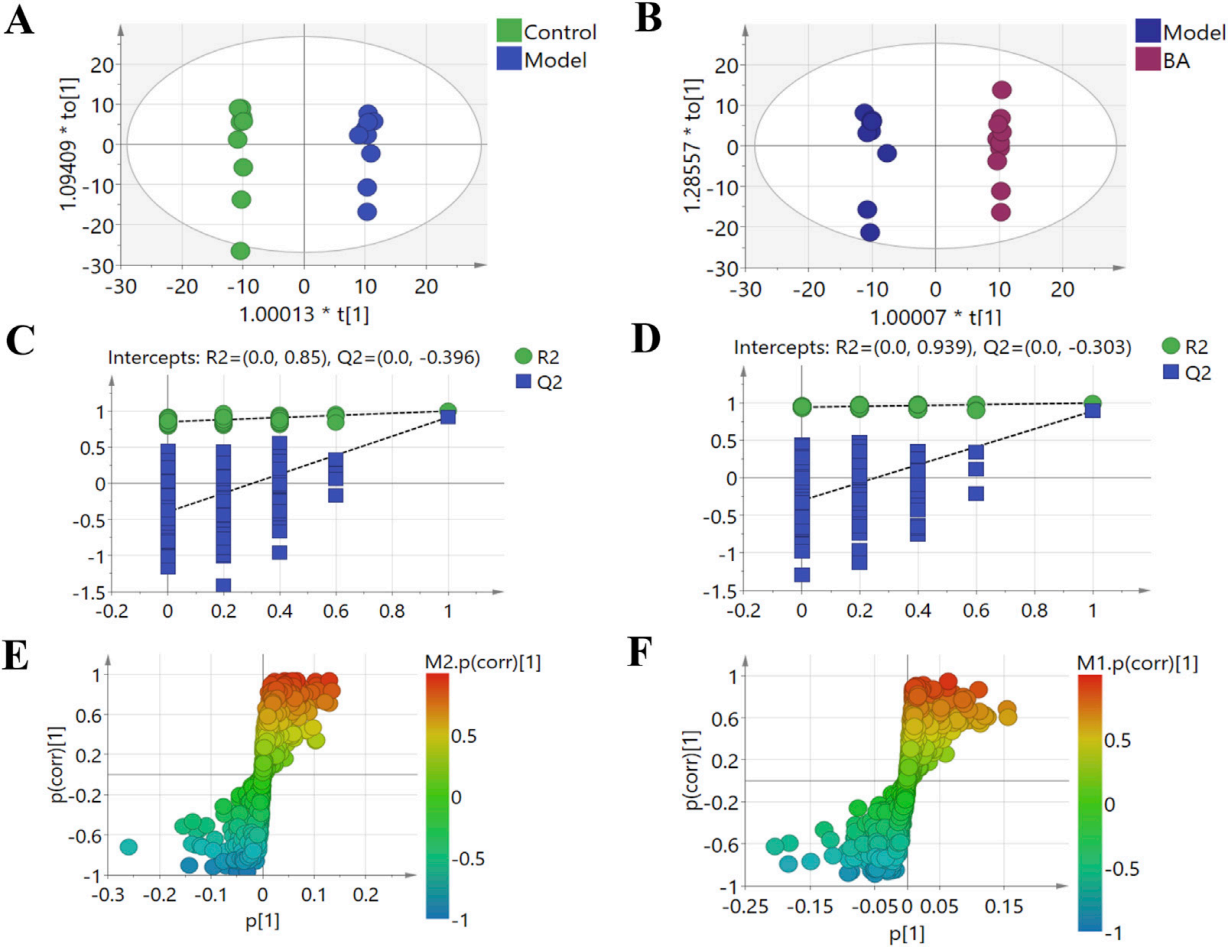
Multivariate statistical analysis of the serum metabolites in the positive (+) ion modes: **(A)** OPLS-DA score plot for the Control group vs. the Model group; **(B)** OPLS-DA score plot for the Model group vs. the BA group; **(C)** Permutation test for the validity of the OPLS-DA model for the Control group vs. the Model group; **(D)** Permutation test for the validity of the OPLS-DA model for the Model group vs. the BA group; **(E)** S-plot obtained from Control and Model groups; **(F)** S-plot obtained from Model and BA groups.

**FIGURE 4:**
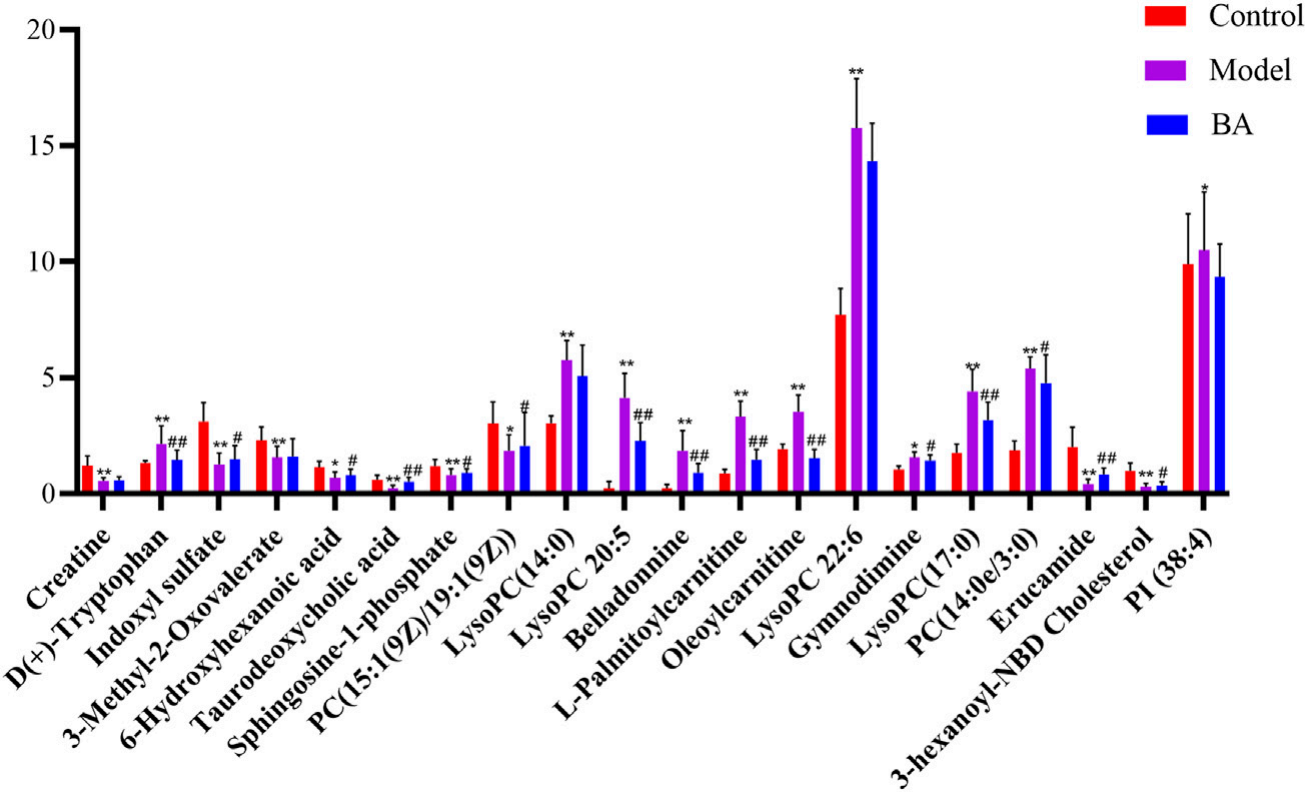
The relative levels of 20 differential metabolites in mice after BA treatment. n = 10 per group. Data are expressed as mean ± SD. Differences were assessed by one-way ANOVA with Dunnett’s method. BA, Bempedoic acid-treated group. Compared with Control group, ^*^ *p* < 0.05, ^**^ *p* < 0.01; Compared with Model group, ^#^ *p* < 0.05, ^##^ *p* < 0.01.

**FIGURE 5:**
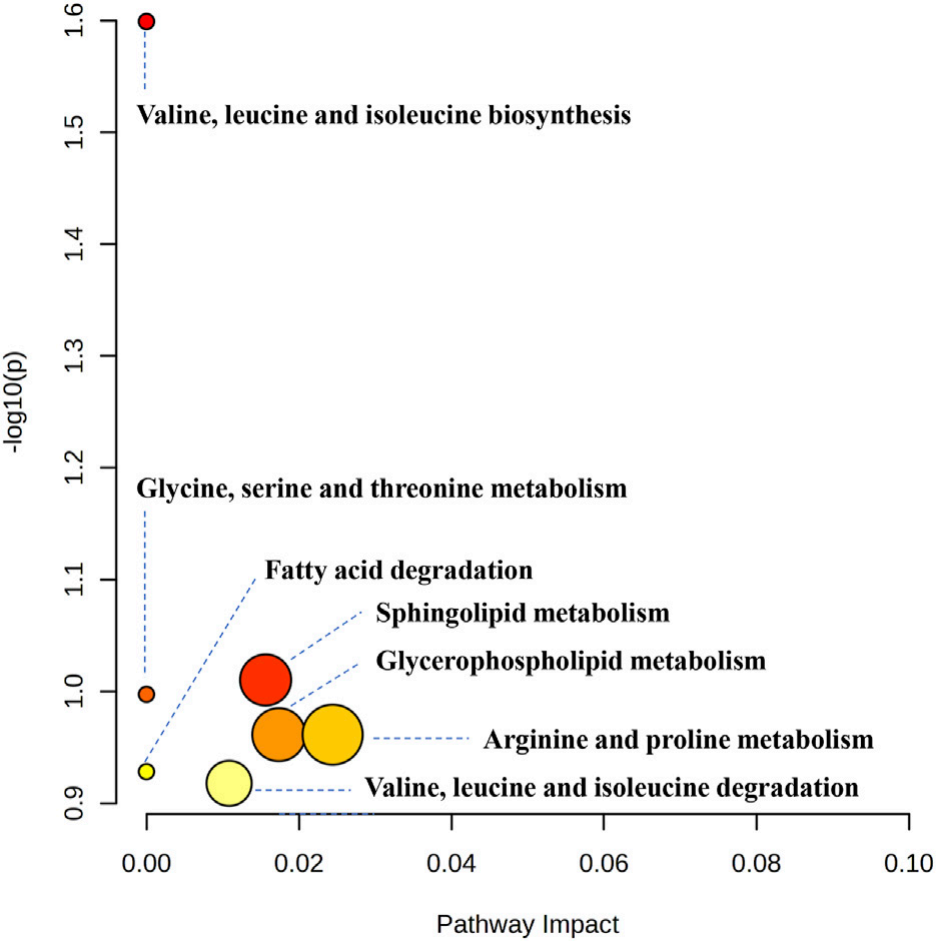
Metabolic pathway analysis results based on the 20 significant regulated serum differential metabolites after BA treatment.

**FIGURE 6:**
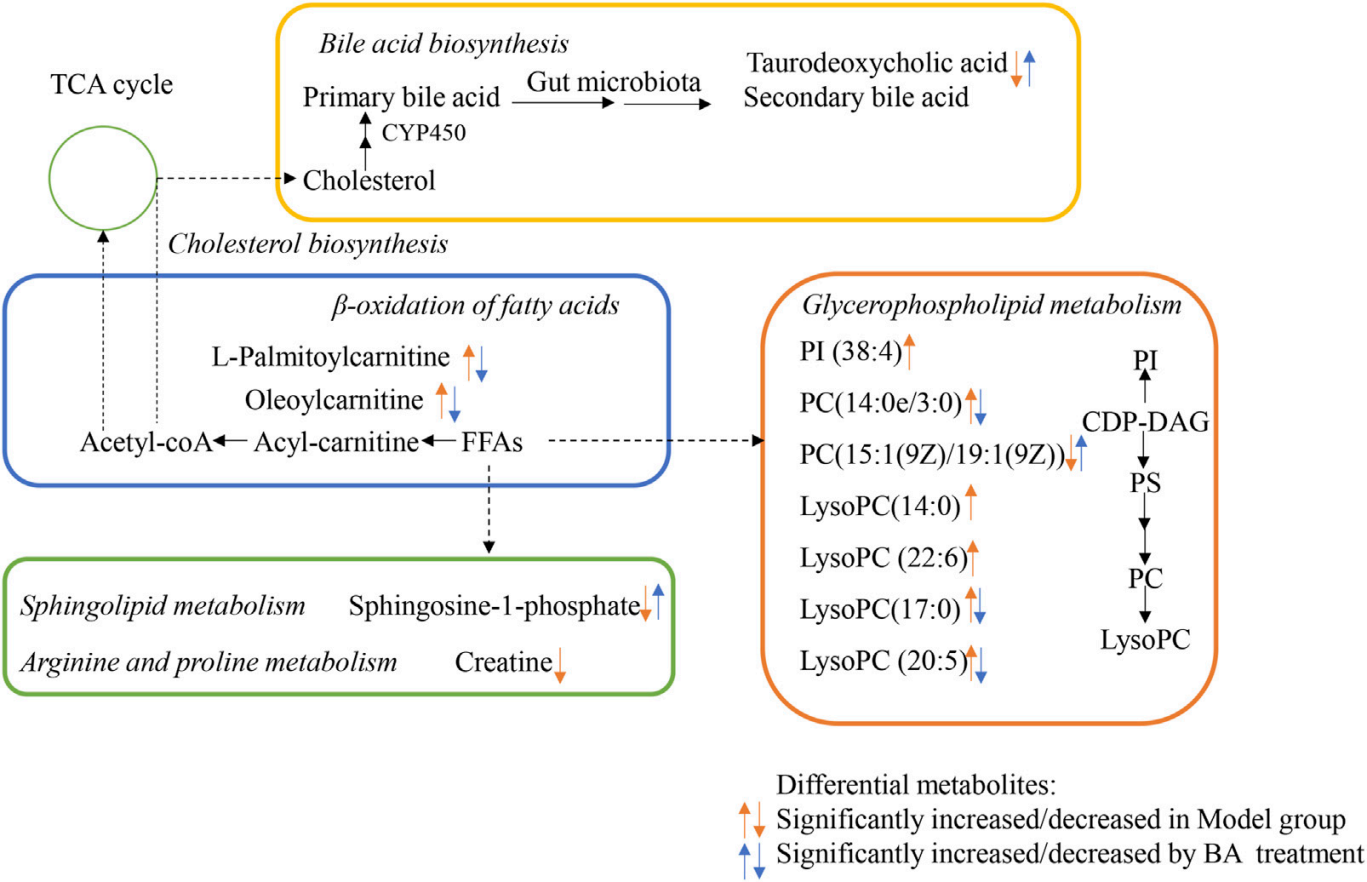
Proposed Metabolic networks of BA on hyperlipidemia.

Using UPLC-Q-TOF-MS/MS, the serum untargeted metabolomics study was conducted to evaluate the effects of BA on gut microbial metabolites in HFD-fed mice. QC samples injected every 8 samples ensured system stability, with >80% of peaks showing RSDs <30%. Serum metabolic profiling in the Control group, Model group, and BA group was assessed by multivariate analysis. A clear separation was observed among the Control, Model and BA groups in OPLS-DA score plots in positive modes (Figures 3A,B) and negative modes (Supplementary Figures S1A,B), suggesting differential metabolic profiles between tested groups. 200-permutation test of OPLS-DA was performed to verify the availability of the model (Figures 3C,D; Supplementary Figures S1C,D). The result of permutation test showed that all the R2 values and Q2 values to the left were lower than their original points to the right, indicating that the OPLS-DA models were valid without overfitting. The significantly changed metabolites between the groups were filtered out based on VIP values >1 (Figures 3E,F; Supplementary Figures S1E,F) and *p* values <0.05 (significance tests). The chemical structures of important metabolites were then identified according to online databases such as HMDB and Chemspider using the data of accurate masses and MS/MS fragments. A total of 20 differential metabolites were identified in the Model and Control groups and significantly regulated by BA treatment (Table 1; Figure 4). And these 20 metabolites were used to figure out the metabolic pathways highly associated with the outcomes of BA treatment using MetaboAnalyst 4.0 and KEGG database. The metabolic pathway analysis revealed that seven pathological processes were closely related to BA treatment, including sphingolipid metabolism, glycerophospholipid metabolism, arginine and proline metabolism, citrate cycle, glycine, serine and threonine metabolism, fatty acid degradation and valine, leucine and isoleucine degradation (Figure 5). Figure 6 summarized the metabolic networks of BA on hyperlipidemia.

**TABLE 1:** 20 differential metabolites in Control, Model and BA-treated groups.

No	Retention time (min)	Matebolitie identification	Molecular formula	Major class	Ion form	Error (ppm	Measured mass (Da)	Content (calibrated intensity)		
								Control	Model	BA
1	1.19	Creatine	C_4_H_9_N_3_O_2_	Guanidino compounds	[M + H]+	4.4	132.0762	1.20 ± 0.43	0.55 ± 0.14^**^	0.59 ± 0.15
2	5.38	D (+)-Tryptophan	C_11_H_12_N_2_O_2_	amino acids	[2M-H]-	1	407.1716	1.33 ± 0.09	2.15 ± 0.78^**^	1.46 ± 0.42^##^
3	5.99	Indoxyl sulfate	C_8_H_7_NO_4_S	aryl sulfates	[M-H]-	0.7	212.0025	3.12 ± 0.82	1.27 ± 0.48^**^	1.48 ± 0.60^#^
4	6.39	3-Methyl-2-Oxovalerate	C_6_H_10_O_3_	α-keto acids	[M-H]-	0.6	129.0555	2.31 ± 0.57	1.56 ± 0.49^**^	1.60 ± 0.77
5	6.60	6-Hydroxyhexanoic acid	C_6_H_12_O_3_	hydroxy fatty acids	[M-H]-	14	131.0713	1.14 ± 0.26	0.68 ± 0.27^*^	0.80 ± 0.26^#^
6	10.17	Taurodeoxycholic acid	C_26_H_45_NO_6_S	bile acids	[M-H]-	2.6	498.2886	0.62 ± 0.19	0.24 ± 0.13^**^	0.53 ± 0.18^##^
7	11.17	Sphingosine-1-phosphate	C_18_H_38_NO_5_	sphingolipids	[M-H]-	2.9	378.2406	1.19 ± 0.30	0.80 ± 0.28^**^	0.90 ± 0.18^#^
8	11.28	PC (15:1 (9Z)/19:1 (9Z))	C_42_H_80_NO_8_P	phosphatidylcholine	[M + H]+	2.3	758.5677	3.02 ± 0.94	1.86 ± 0.68^*^	2.07 ± 1.43^#^
9	11.29	LysoPC (14:0)	C_22_H_46_NO_7_P	lysophospholipids	[M + FA-H]-	0.7	512.2985	3.03 ± 0.32	5.76 ± 0.84^**^	5.06 ± 1.35
10	11.34	LysoPC 20:5	C_28_H_48_NO_7_P	lysophospholipids	[M + H]+	2.7	542.3227	0.24 ± 0.29	4.14 ± 1.05^**^	2.28 ± 0.78^##^
11	11.34	Belladonnine	C_34_H_42_N_2_O_4_	Alkaloids	[M + H]+	8.4	543.3263	0.24 ± 0.16	1.85 ± 0.87^**^	0.92 ± 0.39^##^
12	11.53	L-Palmitoylcarnitine	C_23_H_45_NO_4_	acylcarnitines	[M + H]+	2	400.3414	0.89 ± 0.16	3.32 ± 0.67^**^	1.47 ± 0.43^##^
13	11.73	Oleoylcarnitine	C_25_H_47_NO_4_	acylcarnitines	[M + H]+	2.3	426.3568	1.93 ± 0.21	3.53 ± 0.72^**^	1.53 ± 0.38^##^
14	11.99	LysoPC 22:6	C_30_H_50_NO_7_P	lysophospholipids	[M + FA-H]-	0.2	612.33	7.73 ± 1.13	15.76 ± 2.14^**^	14.34 ± 1.63
15	12.09	Gymnodimine	C_32_H_45_NO_4_	cyclic imine toxins	[M + H]+	7.7	508.3383	1.05 ± 0.16	1.56 ± 0.24^*^	1.44 ± 0.23^#^
16	12.81	LysoPC (17:0)	C_25_H_52_NO_7_P	lysophospholipids	[M + H]+	2.9	510.354	1.76 ± 0.37	4.41 ± 0.96^**^	3.17 ± 0.77^##^
17	12.90	PC (14:0e/3:0)	C_25_H_52_NO_7_P	phosphatidylcholine	[M + FA-H]-	0.6	554.3455	1.87 ± 0.40	5.41 ± 0.50^**^	4.77 ± 1.22^#^
18	16.33	Erucamide	C_22_H_43_NO	fatty acid amides	[M + H]+	1.8	338.3412	2.02 ± 0.85	0.41 ± 0.21^**^	0.83 ± 0.28^##^
19	17.59	3-hexanoyl-NBD Cholesterol	C_39_H_58_N_4_O_5_	lipid analogs	[M + H]+	5.2	663.4515	1.00 ± 0.33	0.30 ± 0.15^**^	0.36 ± 0.16^#^
20	18.68	PI (38:4)	C_47_H_83_O_13_P	glycerophospholipids	[M-H]-	0.4	885.5496	9.89 ± 2.17	10.49 ± 2.52^*^	9.35 ± 1.42

### 3.4 Correlation between gut microbiota and serum metabolite and pathological indexes

**FIGURE 7:**
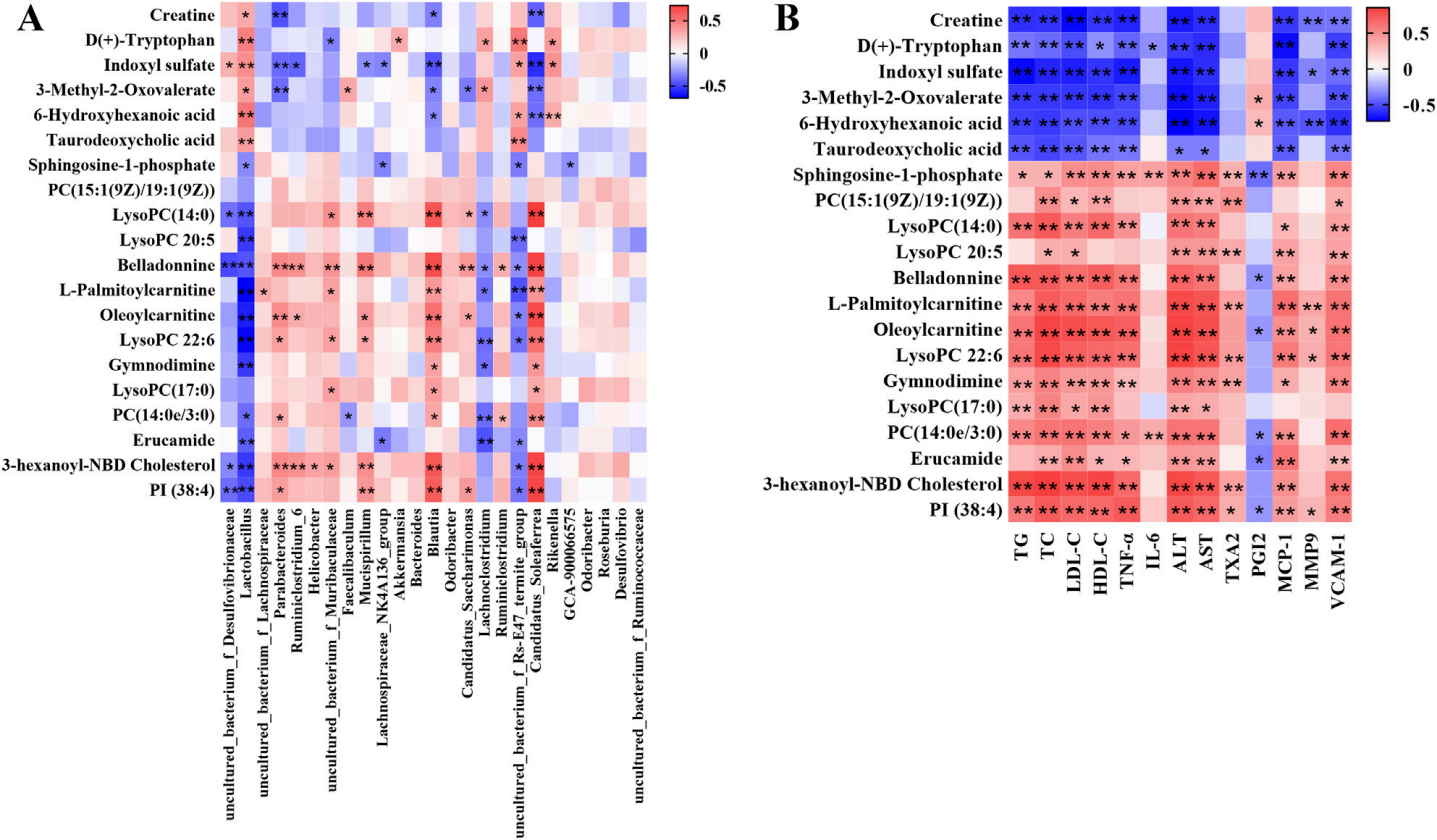
**(A)** Correlation analysis of relative abundances of gut microbiota in the genus level and serum metabolite levels. **(B)** Correlation analysis of serum metabolite levels and pathological indexes. Red means positive correlation, blue means negative correlation, ^*^ *p* < 0.05, ^**^ *p* < 0.01.

In order to comprehensively analyze the relationship among the hyperlipidemia-related indexes, gut microbiota and related metabolites, spearman correlation analysis was performed using the experimental data. As shown in Figure 7A, the covariation between the relative abundances of the gut microbiota and serum metabolite levels are presented in the form of a heatmap diagram. The result reflected that variety of metabolites could be well predicted by the relative abundances of gut microbiota significantly modulated by BA treatment. In addition, Figure 7B showed the covariation between the serum metabolite and pathological indexes.

## 4 Discussion

This study investigated the protective effects of long-term BA administration in HFD-fed mice and performed an integrated analysis of the microbiome and metabolome. The results showed that BA treatment significantly improved blood lipid metabolism, inhibited vascular inflammation, and improved endothelial function, thereby effectively inhibiting hyperlipidemia development. Furthermore, BA restored the gut microbiota imbalance induced by HFD feeding. Key bacterial genera, including *Akkermansia* (enhanced), *Bacteroides* (enhanced), *Roseburia* (enhanced), *Faecalibacterium* (enhanced), and *uncultured_bacterium_f_Muribaculaceae* (enhanced), were closely associated with BA’s protective effects. Moreover, BA ameliorated disturbances in serum metabolite levels, significantly regulating 20 differentially abundant metabolites, mainly including bile acids, acylcarnitines and lysophospholipids. Metabolic pathway analysis revealed these metabolites were associated with pathological processes such as sphingolipid metabolism, glycerophospholipid metabolism, and arginine and proline metabolism.

Hyperlipidemia is a chronic disorder characterized by abnormal lipid metabolism, which represents one of the prominent risk factors for the development of atherosclerosis. Atherosclerosis is chronic pathological process accompanied by the production of a wide range of chemokines, cytokines, and vascular growth factors. In the current study, long-term HFD feeding induced the elevated levels of LDL-C, TC, TG in the Model mice. After treatment with ATO or BA, these indicators dropped remarkably. Endothelial dysfunction is commonly observed in atherosclerotic cardiovascular disease. ATO and BA treatment significantly decreased the TXA2 level while increased the PGI2 level, thereby effectively improving endothelial dysfunction. Hyperlipidemia is known to eventually lead to atherosclerosis. Atherosclerosis-related indicators were also determined in the current study. MCP-1 is one of the important cytokines with the function of chemotaxis and activation of monocytes, which can gather monocytes/macrophages to migrate into the subendothelial layer of the artery, and then the macrophages can form foam cells through uptake of oxidized low-density lipoprotein (ox-LDL) to accelerate the formation of atherosclerotic plaques (Potashnikova et al., 2024). VCAM-1 plays a crucial role in atherosclerosis formation by promoting endothelial injury and the release of various cytokines that stimulate smooth muscle cell transformation and proliferation, leading to fibrous plaque formation and affecting plaque stability (Thayse et al., 2020). MMP9 is closely associated with thin fibrous cap formation, plaque rupture and intraplaque hemorrhage. Downregulation of MMP9 expression in lesions plays an important role in maintaining plaque stability (Li et al., 2020). In the current study, ATO and BA significantly decreased the level of MCP-1 (*p* < 0.01), VCAM-1 (*p* < 0.01) and MMP9 (*p* < 0.05) (Figure 1D), indicating that BA may play a positive role in preventing the formation and development of atherosclerosis.

Multiple studies have explored the role of gut microbiota in hyperlipidemia and identified a strong link between them (Jia et al., 2021; Chen et al., 2022; Lv et al., 2023). In this study, 16S rRNA sequencing was used to investigate the impact of BA on gut microbiota in hyperlipidemia. Alpha diversity analysis revealed a significant increase in fecal microbial diversity after BA treatment. *Firmicutes* and *Bacteroidetes* are the two most dominant phylum at the phylum level, accounting for about 90%. An elevated F/B ratio has been significantly linked to cardiovascular disease (Mariat et al., 2009). Our results showed that BA treatment increased *Bacteroidetes* abundance and decreased *Firmicutes* abundance (Figure 2C), thereby reversing the HFD-induced increase in the F/B ratio. At the genus level, BA treatment significantly increased the abundance of *Akkermansia*, *Bacteroides*, *Roseburia*, *Faecalibacterium* and *Uncultured_bacterium_f_Muribaculaceae* abundance (Figure 2D). *Akkermansia,* a strict anaerobic bacterium from human feces, typically constitutes 1%–3% of the gut microbiota, with propionic acid as its main metabolite (Cani et al., 2022). A series of studies have illustrated that the abundance of *Akkermansia* has a significant negative correlation with various diseases such as cardiovascular diseases, obesity, diabetes, and atherosclerosis (Hasani et al., 2021). *Bacteroides*, a major producer of short-chain fatty acids, is involved in many important metabolic activities in the human colon, including carbohydrate fermentation, nitrogenous substance utilization, and biotransformation of bile acids and other steroids (Flint et al., 2015). Metabolites secreted by *Bacteroides*, such as acetic acid and propionic acid, possess anti-inflammatory effects and help maintain immune system stability (Vo et al., 2019). Furthermore, two main types of *Bacteroides*, *Bacteroides vulgatus* and *Bacteroides dorei* in the gut have been found to attenuate hyperlipidemia and atherosclerosis by reducing gut microbial lipopolysaccharide production (Yoshida et al., 2018). *Roseburia* is a genus that breaks down indigestible carbohydrates and produces butyrate. It serves as a biomarker for conditions like gallstone formation and as a beneficial microbe for probiotic applications. Studies have shown that *Roseburia* has a beneficial effect on hyperlipidemia (Xu et al., 2021; Duan et al., 2024). *Faecalibacterium* is one of the most important symbiotic bacteria with the highest content in human intestinal flora. It is also a butyrate producer, exhibiting anti-inflammatory effects, maintaining bacterial enzyme activity, and protecting the digestive system from intestinal pathogens. Evidence shows that *Faecalibacterium* enrichment can improve insulin resistance and plasma triglyceride levels, contributing to hyperglycemia and hyperlipidemia regulation (Tong et al., 2018). Recently, *uncultured_bacterium_f_Muribaculaceae* has been reported to be negatively correlated with lipid levels (Mu et al., 2020). Therefore, BA’s protective effects against hyperlipidemia might be related to the elevated levels of these gut microbiota.

In the current study, the differential metabolites BA significantly regulated mainly include lysophospholipids, acylcarnitines and bile acids. Lysophospholipids, major serum phospholipids, are considered important cell signaling molecules in cardiovascular and metabolic diseases (Tan et al., 2020). Among them, LysoPCs are inflammatory mediators that regulate endothelial cell proliferation and apoptosis, thereby affecting hyperlipidemia and atherosclerosis development (Slijkhuis et al., 2024). In this study, a series of LysoPCs were significantly increased in the model group compared with the control group, while they were decreased after BA treatment (Figures 4, 6). Although increasing evidence suggests that lysophospholipids participate in inflammation and endothelial injury, their mode of action is still poorly understood.

Bile acids are the end products of cholesterol catabolism, synthesized in the liver and released into the small intestine after meal ingestion (Cariello et al., 2017). They are essential for absorption of dietary lipids and metabolism of cholesterol catabolism. In addition to being involved in the digestive process, bile acids act as important signaling molecules in host-gut microbiota crosstalk, regulating the lipid and glucose metabolism by activating relevant receptors and cellular signaling pathways in the liver and gut (Collins et al., 2023). In the current study, the level of secondary bile acid (taurodeoxycholic acid) was lower in the Model group compared with the Control group. BA treatment restored the abnormal taurodeoxycholic acid level (Figures 4, 6), potentially due to gut microbiota changes. However, the specific mechanism of BA on bile acid metabolism needs further elucidation.

The long-chain acylcarnitine, L-palmitoylcarnitine, has been shown to disrupt vascular endothelial function and decrease nitric oxide synthesis, pathways promoting hyperlipidemia progression and subsequent atherosclerosis (Shen et al., 2022). In this study, a higher level of L-palmitoylcarnitine was observed in the Model group compared with the Control group, while BA treatment decreased this level (Figures 4, 6). Additionally, other long-chain acylcarnitines (e.g., oleoylcarnitine) were increased in the Model group and decreased by BA treatment, suggesting that suggesting BA may enhance fatty acid β-oxidation to regulate glucose and lipid metabolism, possibly via gut microbiota modulation.

Sphingolipids are generally considered critical components of cell membrane architecture. Extensive research in animal and yeast models has revealed that sphingolipids and their metabolites are highly significant bioactive molecules playing pivotal roles in essential signal transduction pathways, including cell growth, differentiation, senescence, and programmed cell death (Santos et al., 2020). Several studies suggest that pharmacological therapies targeting dysfunctional sphingolipid metabolism and/or signaling may have beneficial effects in decreasing the chronic pathology of hyperglycemia and hyperlipidemia (Kasbi-Chadli et al., 2021). Recent attention has focused on the crosstalk between cholesterol and sphingolipid biosynthesis pathways. Decreased sphingosine-1-phosphate levels in hyperlipidemia and atherosclerosis can be an important factor for coronary heart disease development (Alessenko et al., 2018). In this study, sphingosine-1-phosphate level was lower in the Model group compared with the Control group, while BA treatment upregulated its level (Figures 4, 6).

MetaboAnalyst and KEGG databases were used to explore possible BA-regulated metabolic pathways. Figure 6 illustrates metabolic pathways altered by hyperlipidemia and targeted by BA treatment. It highlights sphingolipid metabolism, arginine and proline metabolism, and glycerophospholipid metabolism as significant pathways involved in BA’s therapeutic effects.

In recent years, integrated analysis of gut microbiota alterations associated with metabolic phenotype changes has become an effective strategy for understanding potential drug action mechanisms. To further reveal BA’s mechanism, Spearman’s correlation analysis was performed between gut microbiota genera and serum metabolites, and between serum metabolites and pharmacological indexes (Figure 7). This study demonstrated that microbial and metabolomic remodeling by BA could inhibit hyperlipidemia development. However, current understanding is limited, and further investigations are necessary to confirm the mechanisms linking gut microbiome regulation and metabolic alterations to BA’s effects.

## 5 Conclusion

To sum up, the present study demonstrated that BA treatment could improve the blood lipid metabolism, inhibit vascular inflammation and improve the endothelial function. These beneficial effects of BA may be partly attributed to alterations in gut microbiota composition and serum metabolite levels. This work provided a novel insight to understand the mechanism of BA alleviating hyperlipidemia-related diseases and provide a further reference for the clinical application of BA.

## Data Availability

The datasets presented in this study can be found in online repositories. The names of the repository/repositories and accession number(s) can be found below: https://www.ncbi.nlm.nih.gov/, SRP563800.
