# Supplementary material for: Role of gut microbiota in bempedoic acid against hyperlipidemia: a new candidate target for bempedoic acid on the therapeutic regulation
Source: Front Pharmacol. 2025 Jun 3;16:1584273. doi: 10.3389/fphar.2025.1584273 (PMC12170663; doi:10.3389/fphar.2025.1584273)
Supplement: Supplementary file 1 [file Table1.docx]

Table S1 16S rRNA Sequencing depth data in all samples

| Sample ID | Raw CCS | Clean CCS | Effective  CCS | AvgLen(bp) | Effective(  %) | Feature | Coverage |
| --- | --- | --- | --- | --- | --- | --- | --- |
| A1 | 7,364 | 6,379 | 6,371 | 1,459 | 86.52 | 224 | 0.9938 |
| A10 | 7,508 | 6,545 | 6,531 | 1,462 | 86.99 | 178 | 0.9922 |
| A2 | 7,497 | 6,405 | 6,397 | 1,461 | 85.33 | 206 | 0.9934 |
| A3 | 7,453 | 6,282 | 6,187 | 1,482 | 83.01 | 214 | 0.9896 |
| A4 | 7,461 | 6,593 | 6,570 | 1,465 | 88.06 | 204 | 0.9908 |
| A5 | 7,412 | 6,557 | 6,543 | 1,473 | 88.28 | 109 | 0.996 |
| A6 | 7,507 | 6,267 | 6,240 | 1,461 | 83.12 | 202 | 0.9943 |
| A7 | 7,473 | 6,412 | 6,388 | 1,461 | 85.48 | 170 | 0.9933 |
| A8 | 7,465 | 6,339 | 6,326 | 1,459 | 84.74 | 199 | 0.9911 |
| A9 | 7,391 | 6,379 | 6,350 | 1,459 | 85.92 | 193 | 0.9937 |
| B1 | 7,479 | 6,577 | 6,576 | 1,460 | 87.93 | 259 | 0.9926 |
| B10 | 7,537 | 6,471 | 6,467 | 1,461 | 85.8 | 215 | 0.9917 |
| B2 | 7,506 | 6,626 | 6,620 | 1,459 | 88.2 | 232 | 0.9921 |
| B3 | 7,494 | 6,521 | 6,514 | 1,462 | 86.92 | 213 | 0.9934 |
| B4 | 7,461 | 6,484 | 6,483 | 1,462 | 86.89 | 244 | 0.993 |
| B5 | 7,478 | 6,406 | 6,377 | 1,469 | 85.28 | 177 | 0.9941 |
| B6 | 7,556 | 6,621 | 6,530 | 1,463 | 86.42 | 298 | 0.9914 |
| B7 | 7,510 | 6,498 | 6,489 | 1,458 | 86.4 | 240 | 0.9917 |
| B8 | 7,481 | 6,387 | 6,367 | 1,460 | 85.11 | 231 | 0.9909 |
| B9 | 7,556 | 6,663 | 6,647 | 1,462 | 87.97 | 203 | 0.99 |
| C1 | 7,562 | 6,337 | 6,293 | 1,465 | 83.22 | 188 | 0.9906 |
| C10 | 7,579 | 6,489 | 6,477 | 1,459 | 85.46 | 101 | 0.9954 |
| C2 | 7,525 | 6,627 | 6,625 | 1,451 | 88.04 | 237 | 0.9939 |
| C3 | 7,462 | 6,519 | 6,516 | 1,458 | 87.32 | 198 | 0.9906 |
| C4 | 7,528 | 6,542 | 6,534 | 1,459 | 86.8 | 208 | 0.9913 |
| C5 | 7,463 | 6,490 | 6,488 | 1,457 | 86.94 | 157 | 0.9919 |
| C6 | 7,447 | 6,439 | 6,432 | 1,458 | 86.37 | 232 | 0.9905 |
| C7 | 7,526 | 6,469 | 6,450 | 1,459 | 85.7 | 172 | 0.9908 |
| C8 | 7,459 | 6,442 | 6,442 | 1,456 | 86.37 | 251 | 0.9945 |
| C9 | 7,430 | 6,576 | 6,573 | 1,460 | 88.47 | 199 | 0.993 |
| M1 | 7,404 | 6,507 | 6,505 | 1,458 | 87.86 | 242 | 0.9911 |
| M10 | 7,493 | 6,539 | 6,533 | 1,460 | 87.19 | 220 | 0.9931 |
| M2 | 7,418 | 6,454 | 6,446 | 1,461 | 86.9 | 171 | 0.9919 |
| M3 | 7,496 | 6,578 | 6,570 | 1,469 | 87.65 | 142 | 0.9935 |
| M4 | 7,448 | 6,528 | 6,524 | 1,465 | 87.59 | 191 | 0.9924 |
| M5 | 7,504 | 6,584 | 6,577 | 1,465 | 87.65 | 195 | 0.9912 |
| M6 | 7,449 | 6,575 | 6,565 | 1,477 | 88.13 | 161 | 0.9915 |
| M7 | 7,437 | 6,526 | 6,522 | 1,468 | 87.7 | 165 | 0.9941 |
| M8 | 7,588 | 6,576 | 6,526 | 1,459 | 86 | 115 | 0.9929 |
| M9 | 7,456 | 6,606 | 6,599 | 1,462 | 88.51 | 217 | 0.9939 |

Note: A: Atorvastatin group; B: Bempedoic acid group; C: Control group; M: Model group. “Raw-CCS” refers to the number of CCS （Circular Consensus Sequencing）identified for this sample. “Clean CCS” refers to the number of sequences after identifying and removing primers; “Effective-CCS” refers to the number of sequences used for subsequent analysis after length filtering and chimerism removal; “AvgLen (bp)” refers to the average sequence length of the sample; Effective (%) refers to the percentage of Effective-CCS in Raw-CCS. "Feature" refers to the number of OTUs. "Coverage" indicates the proportion of species that have been detected among the total species. Coverage > 0.99 indicating sufficient sequencing depth.
