# Supplementary figures and images for: Role of gut microbiota in bempedoic acid against hyperlipidemia: a new candidate target for bempedoic acid on the therapeutic regulation
Source: Front Pharmacol. 2025 Jun 3;16:1584273. doi: 10.3389/fphar.2025.1584273 (PMC12170663; doi:10.3389/fphar.2025.1584273)

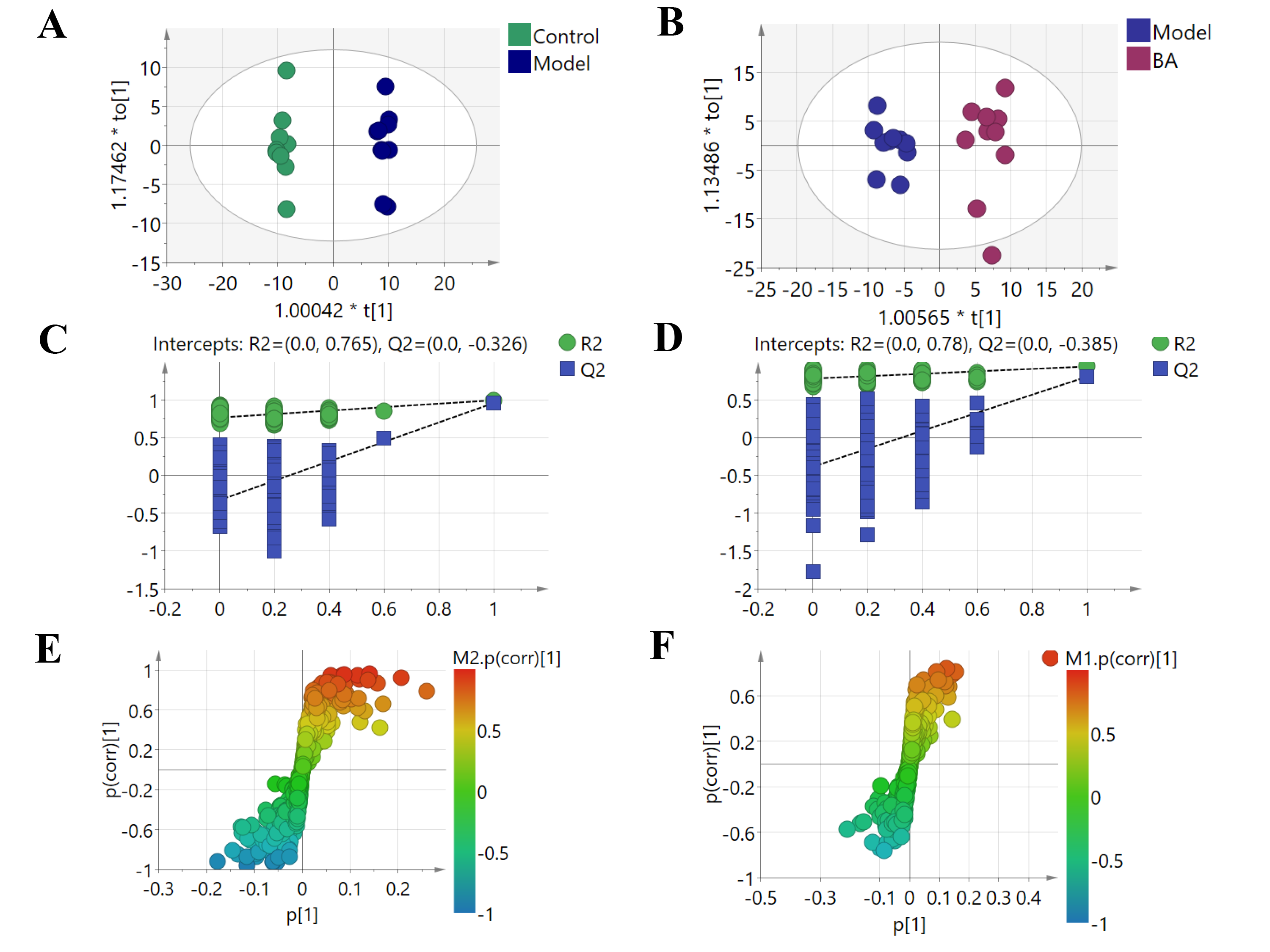

Supplement: Supplementary file 2 [file Image1.tif]
